# Substance use before or during pregnancy and the risk of child mortality, perinatal morbidities and congenital anomalies

**DOI:** 10.1017/S2045796023000549

**Published:** 2023-07-11

**Authors:** Charles Tzu-Chi Lee, Vincent Chin-Hung Chen, Johnny Kuang-Wu Lee, Shu-I Wu, Gillian Cheng, Tzu-Min Kao, Shih-Yuan Wang, Michael Gossop

**Affiliations:** 1 Department of Health Promotion and Health Education, National Taiwan Normal University, Taipei, Taiwan; 2 Department of Psychiatry/Health Information and Epidemiology Laboratory, Chang Gung Memorial Hospital at Chiayi, Puzi, Taiwan; 3 Department of Psychiatry, Chang Gung University, Taoyuan, Taiwan; 4 General Education Center, University of Taipei, Taipei, Taiwan; 5 Department of Medicine, Mackay Medical College, New Taipei, Taiwan; 6 Department of Psychiatry and Suicide Prevention Center, Mackay Memorial Hospital, Taipei, Taiwan; 7 Department of Exercise Health Science, National Taiwan University of Sports, Taichung City, Taiwan; 8 King’s College London, National Addiction Centre, London, UK

**Keywords:** congenital anomalies, mortality, perinatal morbidities, pregnancy, substance

## Abstract

**Aims:**

We aimed to investigate child mortality, perinatal morbidities and congenital anomalies born by women with substance misuse during or before pregnancy (DP or BP).

**Methods:**

Taiwan Birth Registration from 2004 to 2014 linking Integrated Illicit Drug Databases used to include substance misuse participates. Children born by mothers convicted of substance misuse DP or BP were the substance-exposed cohort. Two substance-unexposed comparison cohorts were established: one comparison cohort selected newborns from the rest of the population on a ratio of 1:1 and exact matched by the child’s gender, child’s birth year, mother’s birth year and child’s first use of the health insurance card; another comparison cohort matched newborns from exposed and unexposed mothers by their propensity scores calculated from logistic regression.

**Results:**

The exposure group included 1776 DP, 1776 BP and 3552 unexposed individuals in exact-matched cohorts. A fourfold increased risk of deaths in children born by mothers exposed to substance during pregnancy was found compared to unexposed group (hazard ratio [HR] = 4.54, 95% confidence interval (CI): 2.07–9.97]. Further multivariate Cox regression models with adjustments and propensity matching substantially attenuated HRs on mortality in the substance-exposed cohort (aHR = 1.62, 95% CI: 1.10–2.39). Raised risks of perinatal morbidities and congenital anomalies were also found.

**Conclusions:**

Increased risks of child mortality, perinatal morbidities or congenital anomalies were found in women with substance use during pregnancy. From estimates before and after adjustments, our results showed that having outpatient visits or medical utilizations during pregnancy were associated with substantially attenuated HRs on mortality in the substance-exposed cohort. Therefore, the excess mortality risk might be partially explained by the lack of relevant antenatal clinical care. Our finding may suggest that the importance of early identification, specific abstinence program and access to appropriate antenatal care might be helpful in reducing newborn mortality. Adequate prevention policies may be formulated.

## Introduction

Substance abuse has always been an important public health issue. According to the United Nations Office on Drugs and Crime report, over 280 million people aged between 15 and 64 used at least one illicit drug in the past year, accounting for 5% of the population (United Nations Office on Drugs and Crime, 2017). Most female substance users are of childbearing age. The prevalence of substance use before or during pregnancy may range from 1% to as high as 21% (Australian Institute of Health and Welfare, 2016; Minozzi *et al.*, 2020). The use of substances during pregnancy may increase the risk of mortality, premature birth, low birth weight, nervous system damage or delayed mental development (Ali *et al.*, 2016; Gunn *et al.*, 2016; Oei *et al.*, 2012; Wolfe *et al.*, 2005). Previous small and cross-sectional research described 1.3–4.3 times elevated risks of infant mortality (Fang *et al.*, 2018; Good *et al.*, 2010; Saleh Gargari *et al.*, 2012; Wolfe *et al.*, 2005) and an up to sixfold increased risk of low birth weight, preterm birth or microcephaly (Kivisto *et al.*, 2015; Minozzi *et al.*, 2020; Noland *et al.*, 2005; Sudekum *et al.*, 2019) comparing substance (including methamphetamine, opioid, heroin, methadone or alcohol)-exposed pregnant mothers to unexposed ones.

Past studies on substance-related topics have mostly focused on characteristics or health risks among substance users (Hser *et al.*, 2012). Very few studies were among women during pregnancy. Substances studied in these few research were mostly heroin or methadone and only scarce were on amphetamine (Oei *et al.*, 2012). Most past research had limitations of cross-sectional design that was not able to explore causal relationships, small sample sizes with insufficient numbers to compare rare outcomes or the incapability to control for other mother’s health or disease-related adverse confounders associated with drug-dependence (Oei *et al.*, 2012). In particular, there is still a paucity in literature using long-term population-based data and adequately matched comparisons to examine whether substance exposures (including amphetamine or ketamine) among women during pregnancy were associated with excess risks of mortality, as well as influences on perinatal morbidities, congenital heart (Perumal *et al.*, 2019) or nervous system defects. All the existing literature has the issue of lacking large and representative sample. Taking the advantage of nationwide substance use related datasets composed by various government departments in Taiwan, we aimed to investigate long-term risks of mortality from birth to 13 years of age, perinatal morbidities and congenital anomalies among children born by mothers exposed to substances during or before pregnancy (DP or BP).

## Method

### Study database

The Integrated Illicit Drug Databases (IIDD) database was consolidated in 2015 to the Health and Welfare Data Science Center (HWDSC) of the Statistics Department of the Ministry of Health and Welfare in Taiwan government. The IIDD including police records for substance misuse contained information of crime records, Birth Registration, Household Registration, National Health Insurance Databases, National Mortality Database and the other dozen databases. After integration, the HWDSC provided the research team with a de-identified secondary data for on-site analysis. All data or ID numbers were encrypted, making sure that no access to any personal identifiable information could be gained. Besides research team performed de-identified secondary data analysis under the strict third-party organization’s supervision (HWDSC), all individual data could not bring out the HWDSC. As for the reliability of using criminal records as a proxy measure of substance exposure, research has shown that approximately 50% of incarcerated individuals, including those sentenced for non-drug-related offenses, are believed to meet the diagnostic criteria for drug abuse or dependence (Chandler *et al.*, 2009; Karberg and James, 2005; Mumola and Karberg, 2007), suggesting that it may be a valid proxy measure of substance exposure among individuals in the criminal justice system.

The date of conception is estimated using the birth date and the number of gestational weeks from the Birth Registration and Birth Notification to define the period of pregnancy. The basic demographic information of these mothers was obtained by linkage to the Household Registration files. Mortality Database served as our outcome of mortality. The National Health Insurance Research Database was linked to explore mothers’ health care utilizations and medications prescribed.

### Ethics approval

The Institutional Review Board of National Taiwan Normal University approved this study (No. 202002HM010). Written consent from the study participants was waived because the data were collected from a population-based database of de-identified secondary data. Taiwan Food and Drug Administration, Department of the Ministry of Health and Welfare approved this study to publish by an official document.

### Study subjects

**Figure 1.**
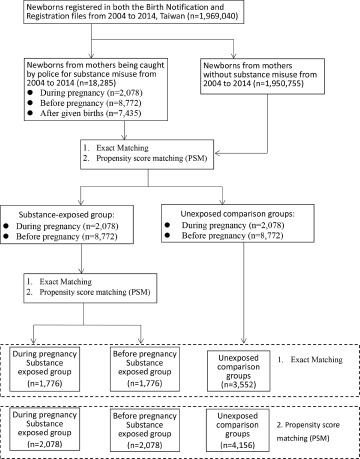
Selection and matching process of study and comparison groups.

This study used Birth Registration and Birth Notification files from 2004 to 2014 and linked to the IIDD that contained police records to identify mothers transferred by police for substance misuse DP or BP. Children born to these mothers were the substance-exposed cohort. A non-substance-exposed comparison cohort was established selecting children from the rest of the population on a ratio of 1:1 and matched by the child’s gender, child’s birth year, mother’s birth year and child’s first use of the health insurance card. To make the characteristics from DP, BP and the unexposed three groups similar for comparisons, and more importantly, we wanted to compare DP vs. the unexposed and BP vs. the unexposed group in the same regression model, a second-stage exact match selecting subjects with similar characteristics from the substance-exposed DP and BP groups to later compare with their exact-matched counterpart was established. For instance, in Fig. 1, we initially identified 2078 DP subjects, but only 1776 found BP subjects with similar characteristics to compare with. Since our main study subjects were DP, we then combined the 1776 unexposed subjects with which DP matched and 1776 unexposed subjects with which BP matched into an exact-matched unexposed cohort (*n* = 3552) for subsequent comparisons.

However, since there were many other parameters of individual characteristics that might be possible confounders, such as mother’s different comorbidities or different medical utilizations (as shown in Table 1, there were still between-group differences after exact matching), it is difficult to select a subset from the comparison cohort that is the same or similar to the DP or BP exposed cohort in terms of various parameters for comparison. To adjust further for such confounding issues and to achieve similarities among substance-exposed DP or BP and unexposed groups, another propensity score (PS)–matched exposed and unexposed cohorts were composed by including subjects that had similar PSs obtained from performing logistic regression on observed covariates (Austin, 2011). The first stage was PS-matching between substance-exposed and unexposed cohorts. The second stage was PS-matching between the substance-exposed DP and BP groups to select subjects with similar characteristics from these two groups (Fig. 1). In Fig. 1, we combined the 2078 unexposed subjects with which DP PS-matched, and 2078 unexposed subjects with which BP PS-matched, into a PS-matched unexposed cohort (*n* = 4156) for subsequent comparisons of the risk of mortality and other outcomes between PS-matched exposed and unexposed cohorts. Children without any use of the health insurance card were excluded.

**Table 1.** Exact-match cohorts, comparisons of subjects’ characteristics of substance-exposed cohort (during pregnancy: *n* = 1,776; before pregnancy: *n* = 1,776) and non-substance-exposed cohort (*n* = 3,552) from exact match by child’s gender, child’s birth year, mother’s birth year and child’s first use of the health insurance card (total sample size for three cohorts = 7,104)

Variable	During pregnancy exposed, *n* (%)	Before pregnancy exposed, *n* (%)	Unexposed, *n* (%)	*p* value
Children, gender				
Male	935 (52.65)	935 (52.65)	1870 (52.65)	>0.99
Female	841 (47.35)	841 (47.35)	1682 (47.35)	
Children, birth year				
2004	103 (5.08)	103 (5.80)	206 (5.80)	>0.99
2005	189 (10.64)	189 (10.64)	378 (10.64)	
2006	205 (11.54)	205 (11.54)	410 (11.54)	
2007	161 (9.07)	161 (9.07)	322 (9.07)	
2008	206 (11.60)	206 (11.60)	412 (11.60)	
2009	147 (8.28)	147 (8.28)	294 (8.28)	
2010	162 (9.12)	162 (9.12)	324 (9.12)	
2011	167 (9.40)	167 (9.40)	334 (9.40)	
2012	124 (6.98)	124 (6.98)	248 (6.98)	
2013	169 (9.52)	169 (9.52)	338 (9.52)	
2014	143 (8.05)	143 (8.05)	286 (8.05)	
Children, first use of the health insurance card				
2004	76 (4.28)	76 (4.28)	152 (4.28)	>0.99
2005	150 (8.45)	150 (8.45)	300 (8.45)	
2006	214 (12.05)	214 (12.05)	428 (12.05)	
2007	152 (8.56)	152 (8.56)	304 (8.56)	
2008	184 (10.36)	184 (10.36)	368 (10.36)	
2009	180 (10.14)	180 (10.14)	360 (10.14)	
2010	165 (9.29)	165 (9.29)	330 (9.29)	
2011	177 (9.97)	177 (9.97)	354 (9.97)	
2012	135 (7.60)	135 (7.60)	270 (7.60)	
2013	154 (8.67)	154 (8.67)	308 (8.67)	
2014	156 (8.78)	156 (8.78)	312 (8.78)	
2015	33 (1.86)	33 (1.86)	66 (1.86)	
Mother, age at this childbirth (year)				
14−17	15 (0.84)	14 (0.79)	28 (0.79)	0.985
18−34	1622 (91.33)	1618 (91.10)	3230 (90.93)	
≥35	139 (7.83)	144 (8.11)	294 (8.28)	
Children, order of this birth				0.135
1	1758 (98.99)	1764 (99.32)	3507 (98.73)	
≥2	18 (1.01)	12 (0.68)	45 (1.27)	
Children, birth place				<0.001
Hospital	1078 (60.70)	1049 (59.07)	2392 (67.34)	
Clinic	662 (37.27)	715 (40.26)	1156 (32.55)	
Other	36 (2.03)	12 (0.68)	4 (0.11)	
Mother, education				<0.001
Elementary, Junior high school	881 (49.61)	774 (43.58)	427 (12.02)	
Senior high school	831 (46.79)	873 (49.16)	1404 (39.53)	
College	64 (3.60)	129 (7.26)	1721 (48.45)	
Mother, marital status				<0.001
Single	595 (33.50)	359 (20.21)	264 (7.43)	
Married	814 (45.83)	1146 (64.53)	3208 (90.32)	
Divorce, widowhood	367 (20.66)	271 (15.26)	80 (2.25)	
Mother, Charlson comorbidity index				<0.001
0	1511 (85.08)	1532 (86.26)	3171 (89.27)	
1	185 (10.42)	189 (10.64)	342 (9.63)	
≥2	80 (4.50)	55 (3.10)	39 (1.10)	
Mother, levels of income				<0.001
<20,000 $NTD	1336 (75.22)	955 (53.80)	547 (15.40)	
20,000−39,999 $NTD	421 (23.70)	755 (42.51)	2216 (62.39)	
≥40,000$NTD	19 (1.07)	66 (3.72)	789 (22.21)	
Mother, residence				<0.001
Rural	460 (25.90)	374 (21.06)	545 (15.34)	
Urban	1316 (74.10)	1402 (78.94)	3007 (84.66)	
Mother, hospital days during pregnancy				
0	171 (9.63)	108 (6.08)	162 (4.56)	<0.001
1−3	699 (39.36)	795 (44.76)	1709 (48.11)	
≥4	906 (51.01)	873 (49.16)	1681 (47.33)	
Mother, outpatient visits during pregnancy				
0−10	813 (45.78)	372 (20.95)	133 (3.74)	<0.001
11−20	494 (27.82)	489 (27.53)	1000 (28.15)	
≥21	469 (26.41)	915 (51.52)	2419 (68.10)	
Mother, prescriptions during pregnancy that are harmful to the fetus				
No	1114 (62.73)	985 (55.46)	2081 (58.59)	<0.001
Yes	662 (37.27)	791 (44.54)	1471 (41.41)	
Mother, prescriptions during pregnancy that are harmful to the fetus in an animal or human experiment				
No	761 (42.85)	638 (35.92)	1274 (35.87)	<0.001
Yes	1015 (57.15)	1138 (64.08)	2278 (64.13)	
Children, Caesarean section				<0.001
No	1148 (64.64)	1069 (60.19)	2389 (67.26)	
Yes	628 (35.36)	707 (39.81)	1163 (32.74)	
Children, fifth minimum APGAR score				<0.001
<7	24 (1.35)	15 (0.84)	9 (0.25)	
≥7	1752 (98.65)	1761 (99.16)	3543 (99.75)	
Children, death				<0.001
No	1756 (98.87)	1766 (99.44)	3543 (99.75)	
Yes	20 (1.13)	10 (0.56)	9 (0.25)	
Perinatal morbidities				<0.001
No	1027 (57.83)	1306 (73.54)	2583 (72.72)	
Yes	745 (41.95)	466 (26.24)	967(27.22)	
Death	4 (0.23)	4 (0.23)	2 (0.05)	
Congenital anomalies				<0.001
No	1660 (93.47)	1680 (94.59)	3373 (94.96)	
Yes	98 (5.52)	88 (4.95)	174 (4.9)	
Death	18 (1.01)	8 (0.45)	5 (0.14)	
Congenital heart diseases				<0.001
No	1725 (97.13)	1738 (97.86)	3481 (98.00)	
Yes	32 (1.80)	29 (1.63)	65 (1.83)	
Death	19 (1.07)	9 (0.51)	6 (0.17)	
Congenital anomalies – spinal cord and other nervous system defects				<0.001
No	1743 (98.14)	1758 (98.99)	3536 (99.55)	
Yes	13 (0.73)	8 (0.45)	7 (0.20)	
Death	20 (1.13)	10 (0.56)	9 (0.25)	
Children, premature birth				<0.001
Pregnancy ≥ 37 week	1364 (76.80)	1547 (87.11)	3241 (91.24)	
Pregnancy < 37 week	412 (23.20)	229 (12.89)	311 (8.76)	
Children, low birth weight				<0.001
≥2500 g	1357 (76.41)	1514 (85.25)	3295 (92.76)	
<2500 g	419 (23.59)	262 (14.75)	257 (7.24)	
Mother, Heroin recidivating				
During pregnancy	863	0	0	
Before pregnancy	787	769	0	
Mother, Amphetamine recidivating				
During pregnancy	931	0	0	
Before pregnancy	775	815	0	
Mother, Ketamine recidivating				
During pregnancy	149	0	0	
Before pregnancy	71	85	0	

### Outcome variables

The main outcomes of this study were mortality and adverse health outcomes from birth to school age (between birth and 13 years of age) in the exposed and unexposed cohorts. Adverse health outcomes included any of the following congenital anomalies: congenital heart diseases (ICD-9-CM: 745–746; ICD-10: Q20–Q24), congenital spinal cord or other nervous (ICD-9-CM: 740–742; ICD-10: Q00–Q07, G90) system defects and perinatal morbidity (ICD-9-CM: 761–763 [fetus or newborn affected by maternal complications of pregnancy, labour, placenta or cord], 764–765 [slow fetal growth, light for dates, fetal growth retardation, low birth weight or prematurity], 767–770 [birth trauma, hypoxia or birth asphyxia], 771–779 [perinatal infections, haemorrhage, hemolysis, endocrine, haematological disorders, digestive system, temperature regulation or convulsions]; ICD-10: P00–P96).

### Covariates

The PS was calculated by including covariates of the child’s gender, child’s birth year, the year of child’s first use of health insurance card, mother’s age at giving birth, birth orders (Wolde *et al.*, 2019), birth place, mother’s education level, mother’s marital status (Institute of Medicine (US) Committee on Understanding Premature Birth and Assuring Healthy Outcomes, 2007; Kafatos and Pantelakis, 1982), Charlson comorbidity index (Bateman *et al.*, 2013), mothers’ levels of income, mother’s urbanization levels (Kafatos and Pantelakis, 1982), mother’s days of hospitalization DP, outpatient visits DP (Bartel *et al.*, 2017), medication prescribed DP (D- and X-grade prescription drugs) (Baldacci *et al.*, 2018) and the method of delivery (Caesarean or natural birth). The premature birth, low birth weight and the physical status at birth (fifth minute APGAR score) (Gaiva *et al.*, 2016) were included in the regression adjustment. The newborn APGAR score is a rapid assessment of the health of newborns. A score of 7–10 points is normal, 4–7 require partial first aid assessment and less than three points require immediate first aid (Baldacci *et al.*, 2018; Kafatos and Pantelakis, 1982).

### Statistical analyses

The study was designed as a retrospective cohort study. Hazards of mortality and health adverse outcomes comparing substance-exposed and -unexposed cohorts were estimated by Cox regression models. This study also included death as a competing risk event for the analysis of perinatal morbidities and congenital anomalies. The starting point was the date of birth, and the study followed these cases to death, immigration or December 31, 2017. Measurements were adjusted in two different models. Model 1 adjusted for the child’s gender, child’s birth year, the year of child’s first use of health insurance card, mother’s age at giving birth, birth orders, birth place, mother’s education level, mother’s marital status, Charlson comorbidity index, mothers’ levels of income, mother’s urbanization levels, mother’s days of hospitalization DP, outpatient visits DP, medication prescribed DP (D- and X-grade prescription drugs) and the method of delivery (Caesarean or natural birth). Model 2 adjusted for covariates in Model 1 and fifth minute APGAR score, premature birth and low birth weight. Analyses were performed by exact-match cohorts and PS-matched cohorts, respectively.

## Results

From 2004 to 2014, there were 1,969,040 newborns in the Birth Registration file, which were then linked to the IIDD that contained police transfer records. There were 18,285 mothers of newborn babies caught by police because of substance misuse between years 2004 and 2014 (Fig. 1).

Among the 18,285 newborns from the substance-exposed cohort, 2,078 and 8,772 newborns were born by mothers exposed to substance DP or BP, respectively. Numbers of 10,850 newborns from unexposed mothers were selected and 1:1 matched by child’s gender, birth year, first use of the health insurance card and mother’s birth year. After the second exact matching, 1,776 subjects from each two substance-exposed subgroups (DP or BP) and 3,552 subjects from the unexposed group were selected. Results from the exact match are shown in Table 1. Child’s gender, birth year, first use of the health insurance card and mother’s birth year were matched well. However, significant differences in most of other covariates were noted. Two stages of PS-matching were performed and matched well in characteristics of substance-exposed cohort and PS-matched substance-unexposed cohorts (Supplementary Table S1).

The mortality rate in the DP group (14.46 [95% confidence interval (CI), 8.83–22.33] per 10,000 person-years) was similar with that in the BP group (6.82 [95% CI, 3.27–12.54] per 10,000 person-years) but higher than the unexposed group (2.99 [95% CI, 1.37–5.68] per 10,000 person-years) during the follow-up period among the exact-matched cohorts. Significant differences were found comparing child mortality, perinatal morbidities, congenital anomalies, prematurity and low birth weight among the exact-matched cohorts. The highest rates were all shown in the subgroup of children born by mothers with substance-exposure DP (all *p* < 0.001, Table 1). Among the PS-matched cohorts, significant differences were also found on rates of child mortality (*p* = 0.023), perinatal morbidities (*p* < 0.001), congenital anomalies (*p* = 0.009), including congenital heart (*p* = 0.027) and nervous system defects (*p* = 0.027), prematurity (*p* < 0.001) and low birth weight (*p* < 0.001, Supplementary Table S1). Causes of death were mainly accidents and injuries (ICD-10: S00-T98, V01-Y98), followed by unknown causes (ICD-10: R00-R99), and morbidities of the perinatal period or congenital malformations (ICD-10: P00-P96, Q00-Q99).

**Figure 2.**
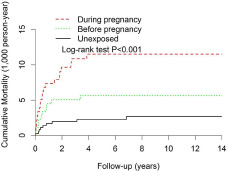
Child mortality analysis of substance-exposed cohort (during pregnancy: *n* = 1,776; before pregnancy: *n* = 1,776) and non-substance-exposed cohort (*n* = 3,552) from exact match by child’s gender, child’s birth year, mother’s birth year and child’s first use of the health insurance card.

Table 2 and Fig. 2 show comparisons of mortality risks between substance-exposed DP, BP and exact-matched unexposed groups by Cox regression analysis. Substance use DP significantly affected child mortality risks (HR = 4.54, 95% CI: 2.07–9.97) (see Table 2). Risks of mortality attenuated after adjusting for demographic variables, mother’s healthcare utilizations, method of delivery in adjustment Model 1 and adding the fifth minute APGAR score, prematurity and low birth weight in Model 2. Other significant outcomes related to substance exposure DP from exact-matched comparisons were increased risks of perinatal morbidities (attenuated HR [aHR] = 1.85, 95% CI: 1.11–3.10) and congenital spinal cord or other nervous system defects (aHR = 4.20, 95% CI: 1.32–13.41) (Table 2). Substance-exposure BP was also shown to have raised risk of perinatal morbidity after adjustment (aOR = 1.38, 95% CI: 1.25–1.54) (Table 2).

**Table 2.** Competing risk-adjusted Cox regression analysis of child’s mortality, perinatal morbidities and congenital anomalies of three exact-match cohorts by child’s gender, child’s birth year, mother’s birth year and the year of child’s first use of health insurance card, *n* = 7,104

		Unadjusted analysis		Adjusted Model 1		Adjusted Model 2	
Variable	Exposed period of pregnancy	HR/OR (95% CI)	*p* value	HR/OR (95% CI)	*p* value	HR/OR (95% CI)	*p* value
Mortality	Unexposed	1.00		1.00		1.00	
	During	4.54 (2.07−9.97)	<0.001	2.17 (0.82−5.75)	0.121	1.94 (0.72−5.26)	0.192
	Before	2.24 (0.91−5.50)	0.080	1.23 (0.46−3.29)	0.676	1.09 (0.40−2.98)	0.860
Perinatal morbidities	Unexposed	1.00		1.00		1.00	
	During	1.58 (1.46−1.70)	<0.001	1.85 (1.11−3.10)	0.019	1.85 (1.11−3.10)	<0.001
	Before	0.78 (0.72−0.84)	<0.001	1.38 (1.25−1.54)	<0.001	1.38 (1.25−1.54)	<0.001
Congenital anomalies	Unexposed	1.00		1.00		1.00	
	During	1.16 (0.90−1.48)	0.253	1.27 (0.92−1.74)	0.142	1.12 (0.81−1.55)	0.495
	Before	1.02 (0.79−1.32)	0.876	1.15 (0.85−1.55)	0.377	1.09 (0.80−1.48)	0.602
Congenital heart diseases	Unexposed	1.00		1.00		1.00	
	During	1.00 (0.65−1.53)	0.996	1.13 (0.64−1.99)	0.678	1.00 (0.56−1.77)	0.996
	Before	0.90 (0.58−1.39)	0.626	1.11 (0.65−1.87)	0.704	1.04 (0.60−1.78)	0.897
Congenital anomalies – spinal cord and other nervous system	Unexposed	1.00		1.00		1.00	
	During	3.78 (1.51−9.49)	0.005	5.79 (1.75−19.12)	0.004	4.20 (1.32−13.41)	0.015
	Before	2.30 (0.83−6.35)	0.108	3.41 (1.01−11.48)	0.048	2.92 (0.83−10.23)	0.094

Adjusted analysis model 1: adjusted for child’s gender, child’s birth year, the year of child’s first use of health insurance card, mother’s age at giving birth, birth orders, birth place, mother’s education level, mother’s marital status, Charlson comorbidity index, mothers’ levels of income, mother’s urbanization levels, mother’s days of hospitalization during pregnancy, outpatient visits during pregnancy, medication prescribed during pregnancy (D- and X-grade prescription drugs), the method of delivery (Caesarean or natural birth) and mortality.

Adjusted analysis model 2: model 1 + the fifth minute APGAR score, premature birth and low birth weight.

HR: hazard ratio; OR: odds ratio.

Perinatal morbidities were compared using logistic regression and reported in OR values.

Cox regression and HR values were compared for the rest of the outcomes.

Cox regression models were also performed to compare the risk of mortality between substance-exposed (DP or BP) and PS-matched unexposed groups (Table 3). Newborn mortality was significantly associated with substance-exposure DP before and after adjustments of demographic variables, mother’s healthcare utilizations, the fifth minute APGAR score, prematurity and low birth weight (aHR = 1.62, 95% CI:1.10–2.39). Significantly increased risk of perinatal morbidity was found in mothers with substance-exposure DP in adjusted Model 1 and those with exposure BP in Model 2. Comparing results of Cox regression analyses that used exact matching (Table 2) to those with PS-matching adjustments Model 2 (Table 3) between pregnant women with or without substance exposure, the most different outcome was found on risks of congenital spinal cord and other nervous system defects using these two different matching methods. A significantly elevated risk was found in women exposed to substance DP using the exact-match method and not those using PS-matching method.

**Table 3.** Competing risk-adjusted Cox regression analysis of child’s mortality, perinatal morbidities and congenital anomalies of three propensity score matched cohorts, *n* = 8,312

		Adjusted Model 1		Adjusted Model 2	
Variable	Exposed period of pregnancy	HR/OR (95% CI)	*p* value	HR/OR (95% CI)	*p* value
Mortality	Unexposed	1.00		1.00	
	During	1.70 (1.16−2.49)	0.007	1.62 (1.10−2.39)	0.015
	Before	1.46 (0.98−2.17)	0.062	1.38 (0.93−2.07)	0.114
Perinatal morbidities	Unexposed	1.00		1.00	
	During	1.32 (1.23−1.41)	<0.001	0.93 (0.65−1.33)	0.693
	Before	0.91 (0.84−0.97)	0.008	1.29 (1.19−1.41)	<0.001
Congenital anomalies	Unexposed	1.00		1.00	
	During	0.96 (0.77−1.21)	0.748	0.87 (0.69−1.10)	0.240
	Before	0.82 (0.65−1.04)	0.107	0.80 (0.63−1.03)	0.079
Congenital heart diseases	Unexposed	1.00		1.00	
	During	0.81 (0.51−1.27)	0.358	0.78 (0.49−1.23)	0.277
	Before	0.79 (0.50−1.24)	0.299	0.77 (0.49−1.22)	0.273
Congenital anomalies –spinal cord and other nervous system	Unexposed	1.00		1.00	
	During	1.75 (0.83−3.68)	0.141	1.36 (0.64−2.86)	0.421
	Before	1.60 (0.75−3.43)	0.223	1.45 (0.67−3.12)	0.348

Adjusted analysis model 1: Propensity score matching for child’s gender, child’s birth year, the year of child’s first use of health insurance card, mother’s age at giving birth, birth orders, birth place, mother’s education level, mother’s marital status, Charlson comorbidity index, mothers’ levels of income, mother’s urbanization levels, mother’s days of hospitalization during pregnancy, outpatient visits during pregnancy, medication prescribed during pregnancy (D- and X-grade prescription drugs) and the method of delivery (Caesarean or natural birth).

Adjusted analysis model 2: regression controlling of fifth minute APGAR score, premature birth and low birth weight.

HR: hazard ratio; OR: odds ratio.

Perinatal morbidities were compared using logistic regression and reported in OR values.

Cox regression and HR values were compared for the rest of the outcomes.

## Discussion

The novelty of this study is the use of a large nationwide population-based cohort data to compare risks of child mortality, congenital anomalies and perinatal morbidities in children born by mothers with or without heroin, amphetamine and ketamine exposures during vs. before pregnancy. The exact and propensity-matched comparisons provided further adjustments for more potential confounders than past studies. Hence, main additions of this study to existing evidence were the long-term approach to follow-up on outcomes of mortality or other developmental diseases using a large population-based representative cohort data, the inclusion of more substances, comparisons between DP vs. BP, DP vs. unexposed and BP vs. unexposed, and more covariates with strict matching methods to adjust for potential confounders that have not been able to be controlled before. Results from exact and PS-matched comparisons revealed elevated risks of mortality and perinatal morbidities among mothers who used substances DP and/or BP. A higher risk of congenital nervous system defects was found among substance-exposure DP mothers than the exact-matched unexposed group. Adjustments or PS-matching by mother’s social economic status, birth years, age at birth, comorbidity index, antenatal outpatient visits or medical expenses substantially attenuated the HRs on mortality in the substance-exposed cohort.

We found excess mortality and perinatal morbidities in children born by mothers with substance exposures DP after PS-matching (and not in the exact-matching comparisons). This finding was consistent with previous research reporting higher mortality in fetus or children born by mothers exposed to opioid, heroin, amphetamine, methamphetamine or methadone compared to that from the general population (Fang *et al.*, 2018; Gorman *et al.*, 2014; Saleh Gargari *et al.*, 2012). Our results showed that the harm of substance use DP had a greater impact than BP, indicating possible effects of intrauterine substance exposures on mortality or perinatal morbidities. Relevant mechanisms for such excess mortality in children born by mothers with intrauterine exposure may be associated with heroin-related neonatal abstinence syndrome, perinatal morbidities (Jones and Fielder, 2015), sudden infant death (Minozzi *et al.*, 2020), preterm labour (Cordeaux *et al.*, 2008; Oei *et al.*, 2012) or amphetamine-induced vasoconstrictions that impair placental perfusion (Cohen *et al.*, 2017), cause placental abruption, fetal growth retardation (Ananth and Vintzileos, 2008) or perinatal deaths (Nijman *et al.*, 2016).

Compared to results from exact-match controls, the elevated risk of mortality decreased from 4-folds to 1.6-folds in the PS-matched cohorts. Such risks remained within similar ranges after further adjustments. Since the PS-matched cohorts controlled for more potential confounders than the exact-matched cohort, the statistical power of PS-matched cohort analysis is larger than the exact-matched analysis. Our finding therefore suggested that when comparing mothers with more similar comorbidities, psychosocial conditions or antenatal medical utilizations after PS-matching, the risk of death would not be overestimated. It may also indicate that improving mother’s access to clinical attention or prenatal care may help decrease child mortality. Literature has reported that pregnant amphetamine users were significantly less likely to receive antenatal care than the general population (Smith *et al.*, 2006). Thus, early identification of pregnant substance users and specific program for abstinence or proper antenatal care should be advised (Oei *et al.*, 2010).

We reported raised perinatal morbidities in children born by mothers with substance use DP or BP compared to exact-matched counterparts. However, after PS-matching and added low birth weight, preterm births and fifth minute APGAR score into the adjustment model, substance use DP no longer lead to significantly increased risks of perinatal morbidities. This finding may suggest the importance of recognizing and managing risk factors for prematurity and low birthweight. Literature has consistently reported that amphetamine or heroin exposures DP were associated with premature delivery or lower birth weight compared to general newborns (Hulse *et al.*, 1997; Oei *et al.*, 2010, 2012; Smith *et al.*, 2006; Vucinovic *et al.*, 2008). Multiple factors including potential polysubstance abuse, maternal nutrition problems or psychosocial stressors, as well as lacking proper assessment measures or study designs may be associated with unknown mechanisms of intrauterine fetal growth retardation (Bell and Lau, 1995; Oei *et al.*, 2012; Vucinovic *et al.*, 2008). Further explorations are still warranted to clarify relevant mechanisms (Oei *et al.*, 2012).

After adjusting for fifth minute APGAR score, premature birth and low birth weight, significantly increased risk of perinatal morbidity was found only in the subgroup of mothers with substance exposure BP and not those with DP. It is possible that the raised risk of perinatal morbidities in these children was also more likely to be related to mother’s domestic stress, social or economic conditions or even the maternal physical or psychiatric care in prison during gestation, which we were not able to control for. Adequate interventions or programs for such psychosocial conditions after the identification of pregnant substance users may be suggested.

In our results, although higher risk of congenital spinal cord or other nervous system defects was found among children born by mothers with substance exposure DP compared to their exact-matched counterparts, no significantly higher risks of any congenital heart or brain anomalies were found when comparing PS-matched groups. Such finding was in line with previous research concluding that intrauterine substance exposure–induced teratogenic risks were not significant (Oei *et al.*, 2012). Some literature compared children born to heroin-dependent mothers separated by early adoption or being raised at home and suggested that intrauterine heroin exposure influenced less than home environments on children’s achievements or other developmental outcomes (Ornoy *et al.*, 1996). Hence, as mentioned above, psychosocial or environmental risk factors among mothers having substance use disorders and their children still requires public health and clinical attention.

### Strengths and limitations

This large population-based cohort study found that exposures to substances DP or BP were associated with increased risk of child mortality, congenital anomalies and perinatal morbidities. Major strengths of this study included careful linkages to several nationwide databases large enough to provide sufficient statistical power. The identification of mothers having substance use was from police records and courts’ sentences with clear confirmation of substance abuse being convicted. Two stages of exact-match and PS-matched multivariate regression models not only supplemented further adjustments of potential confounding variables but also provided more similar distributions on characteristics among the three cohorts. Comparisons of both the exact match and PS-matching in our study demonstrated that PS-matching did not over-pair potential confounders or mediators.

Key limitations would be the lack of other risk factors that were not available in the dataset, such as smoking status, alcohol use or other residual confounders associated with substance use and mortality. Therefore, the possibility and impacts of prenatal polysubstance misuse should still be considered. Second, since the study sample was drawn from police criminal records, it is possible that these substance users only accounted for a small percentage of real users. Being transferred by police for substance misuse can be an indicator of substance exposure to some degree, but it is important to keep in mind that it is not a comprehensive or definitive measure of an individual’s substance use patterns, amount or frequency, the aspects of exposure, level of dependence or overall health and wellbeing. Also, law enforcement data may not capture the full extent of substance use in the population, particularly among those who do not come into contact with the criminal justice system (Hser *et al.*, 2004). Although selection bias and underestimation of the true numbers might exist, we have included substance exposure BP mothers into the three cohort comparisons to improve the credibility. It is important to consider multiple sources of data and use with caution when interpreting law enforcement data as a proxy measure of substance exposure. Third, this study is an observational study and could only provide statistical evidence to examine the hypothesis that mothers’ substance use DP or BP was associated with increased risk of child mortality or perinatal morbidities. Direct biological mechanisms remain to be investigated. However, this study made use of multiple databases, matched and adjusted potential confounders and provided empirical significances from a naturalistic clinical environment.

## Conclusions

In our study, we found elevated risks of child mortality, perinatal morbidities and congenital anomalies born by mothers exposed to substances DP. Such findings may be partially explained by intrauterine substance exposures, as well as mothers’ demographic, psychosocial conditions or degree of prenatal medical care. The worrying phenomenon indicates the need of early identification, specific abstinence program and access to appropriate antenatal care. Provisions of health promotion programs on educating women with substance use ways of protecting maternal and securing fetal physical conditions are also required.

## Data Availability

The data that support the findings of this study are available from the Birth Registration, Birth Notification files, Integrated Illicit Drug Databases and National Health Insurance Research Database provided by the Health and Welfare Data Science Center, Ministry of Health and Welfare (MHW). Academic researchers in Taiwan would need to submit an application to the MHW to have access to these data.

## Supplementary material

For supplementary material accompanying this paper visit http://doi.org/10.1017/S2045796023000549. click here to view supplementary material
