# Supplementary material for: Substance use before or during pregnancy and the risk of child mortality, perinatal morbidities and congenital anomalies
Source: Epidemiol Psychiatr Sci. 2023 Jul 11;32:e43. doi: 10.1017/S2045796023000549 (PMC10387448; doi:10.1017/S2045796023000549)
Supplement: Supplementary file 1 [file S2045796023000549sup001.docx]

Supplementary Table 1. **Propensity score matching (PSM) cohorts,** comparisons of subjects’ characteristics after PSM between substance-exposed cohort during (n=2,078) and before (n=2,078) pregnancy and ‘non-substance-exposed cohort (n=4,156)’ (total sample size for three cohorts=8,312)

| Variable | | | | During pregnancy exposed, n (%) | Before pregnancy exposed, n (%) | Unexposed, n (%) | P value |
| --- | --- | --- | --- | --- | --- | --- | --- |
| Children, gender | | | |  |  |  |  |
| Male | | | | 1081 (52.02) | 1072 (51.59) | 2177 (52.38) | 0.837 |
| Female | | | | 997 (47.98) | 1006 (48.41) | 1979 (47.62) |  |
| Children, birth year | | | |  |  |  |  |
| 2004 | | | | 157 (7.56) | 157 (7.56) | 314 (7.56) | >0.99 |
| 2005 | | | | 240 (11.55) | 240 (11.55) | 480 (11.55) |  |
| 2006 | | | | 258 (12.42) | 258 (12.42) | 516 (12.42) |  |
| 2007 | | | | 197 (9.48) | 197 (9.48) | 394 (9.48) |  |
| 2008 | | | | 223 (10.73) | 223 (10.73) | 446 (10.73) |  |
| 2009 | | | | 168 (8.08) | 168 (8.08) | 336 (8.08) |  |
| 2010 | | | | 188 (9.05) | 188 (9.05) | 376 (9.05) |  |
| 2011 | | | | 185 (8.90) | 185 (8.90) | 370 (8.90) |  |
| 2012 | | | | 135 (6.50) | 135 (6.50) | 270 (6.50) |  |
| 2013 | | | | 178 (8.57) | 178 (8.57) | 356 (8.57) |  |
| 2014 | | | | 149 (7.17) | 149 (7.17) | 298 (7.17) |  |
| Children, first use of the health insurance card | | | | | | | |
| 2004 | | | | 95(4.57) | 97(4.67) | 225(10.83) | 0.746 |
| 2005 | | | | 172 (8.40) | 167 (8.15) | 367 (8.93) |  |
| 2006 | | | | 243 (11.87) | 229 (11.18) | 432 (10.51) |  |
| 2007 | | | | 181 (8.84) | 198 (9.66) | 401 (9.75) |  |
| 2008 | | | | 211 (10.30) | 219 (10.69) | 428 (10.41) |  |
| 2009 | | | | 215 (10.50) | 207 (10.10) | 411 (10.00) |  |
| 2010 | | | | 179 (8.74) | 204 (9.96) | 386 (9.39) |  |
| 2011 | | | | 218 (10.64) | 180 (8.78) | 417 (10.14) |  |
| 2012 | | | | 151 (7.37) | 172 (8.39) | 311 (7.57) |  |
| 2013 | | | | 169 (8.25) | 181 (8.83) | 338 (8.22) |  |
| 2014 | | | | 168 (8.20) | 149 (7.27) | 323 (7.86) |  |
| 2015 | | | | 46(2.21) | 46(2.21) | 64(3.08) |  |
| Mother, age at this childbirth (year) | | | | | | | |
| 14-17 | | | | 32 (1.54) | 23 (1.11) | 162 (3.90) | <0.001 |
| 18-34 | | | | 1829 (88.02) | 1848 (88.93) | 3353 (80.68) |  |
| ≥35 | | | | 217 (10.44) | 207 (9.96) | 641 (15.42) |  |
| Children, order of this birth | | | |  |  |  |  |
| 1 | | | | 2060 (99.13) | 2068 (99.52) | 4117 (99.06) | 0.154 |
| ≥2 | | | | 18 (0.87) | 10 (0.48) | 39 (0.94) |  |
| Children, birth place | | | |  |  |  |  |
| Hospital | | | | 1251 (60.20) | 1209 (58.18) | 2530 (60.88) | 0.004 |
| Clinic | | | | 787 (37.87) | 850 (40.90) | 1550 (37.30) |  |
| Other | | | | 40 (1.92) | 19 (0.91) | 76 (1.83) |  |
| Mother, education | | | |  |  |  |  |
| Elementary, Junior high school | | | | 1052 (50.63) | 1081 (52.02) | 2079 (50.02) | 0.012 |
| Senior high school | | | | 953 (45.86) | 930 (44.75) | 1982 (47.69) |  |
| College | | | | 73 (3.51) | 67 (3.22) | 95 (2.29) |  |
| Mother, marital status | | | |  |  |  |  |
| Single | | | | 678 (32.63) | 654 (31.47) | 1347 (32.41) | 0.826 |
| Married | | | | 949 (45.67) | 977 (47.02) | 1941 (46.70) |  |
| Divorce, widowhood | | | | 451 (21.70) | 447 (21.51) | 868 (20.89) |  |
| Mother, Charlson comorbidity index | | | | | | | |
| 0 | | | | 1784 (85.85) | 1824 (87.78) | 3590 (86.38) | 0.409 |
| 1 | | | | 205 (9.87) | 180 (8.66) | 405 (9.74) |  |
| ≥2 | | | | 89 (4.28) | 74 (3.56) | 161 (3.87) |  |
| Mother, level of income | | | |  |  |  |  |
| < 20,000 $NTD | | | | 1564 (75.26) | 1563 (75.22) | 3243 (78.03) | 0.012 |
| 20,000-39,999 $NTD | | | | 494 (23.77) | 491 (23.63) | 888 (21.37) |  |
| ≥40,000$NTD | | | | 20 (0.96) | 24 (1.15) | 25 (0.60) |  |
| Mother, residence | | | |  |  |  |  |
| Rural | | | | 531 (25.55) | 515 (24.78) | 1099 (26.44) | 0.352 |
| Urban | | | | 1547 (74.45) | 1563 (75.22) | 3057 (73.56) |  |
| Mother, hospital days during pregnancy | | | | | | | |
| 0 | | | | 209 (10.06) | 217 (10.44) | 420 (10.11) | 0.884 |
| 1-3 | | | | 820 (39.46) | 846 (40.71) | 1667 (40.11) |  |
| ≥4 | | | | 1049 (50.48) | 1015 (48.85) | 2069 (49.78) |  |
| Mother, outpatient visits during pregnancy | | | | | | | |
| 0-10 | | | | 992 (47.74) | 979 (47.11) | 1895 (45.60) | 0.036 |
| 11-20 | | | | 580 (27.91) | 616 (29.64) | 1156 (27.82) |  |
| ≥21 | | | | 506 (24.35) | 483 (23.24) | 1105 (26.59) |  |
| Mother, prescriptions during pregnancy that are harmful to the fetus | | | | | | | |
| No | | | | 1309 (62.99) | 1313 (63.19) | 2704 (65.06) | 0.171 |
| Yes | | | | 769 (37.01) | 765 (36.81) | 1452 (34.94) |  |
| Mother, prescriptions during pregnancy that are harmful to the fetus in an animal or human experiment | | | | | | | |
| No | | | | 884 (42.54) | 858 (41.29) | 1882 (45.28) | 0.006 |
| Yes | | | | 1194 (57.46) | 1220 (58.71) | 2274 (54.72) |  |
| Children, caesarean section | | | |  |  |  |  |
| No | | | | 1345 (64.73) | 1345 (64.73) | 2708 (65.16) | 0.918 |
| Yes | | | | 733 (35.27) | 733 (35.27) | 1448 (34.84) |  |
| Children, 5^th^ minimum APGAR score | | | | | | | |
| <7 | | | | 32 (1.54) | 31 (1.49) | 57 (1.37) | 0.852 |
| ≥7 | | | | 2046 (98.46) | 2047 (98.51) | 4099 (98.63) |  |
| Children, death | | | |  |  |  |  |
| No | | | | 2030 (97.69) | 2036 (97.98) | 4098 (98.60) | 0.023 |
| Yes | | | | 48 (2.31) | 42 (2.02) | 58 (1.40) |  |
| Perinatal morbidities | | | | | | | |
|  | No | | | 1218 (58.61) | 1392 (66.99) | 2872 (69.10) | <0.001 |
|  | Yes | | | 841 (40.47) | 663 (31.91) | 1255 (30.20) |  |
|  | Death | | | 19 (0.91) | 23 (1.11) | 29 (0.70) |  |
| Congenital anomalies | | | | | | | |
| No | | | | 1924 (92.59) | 1945 (93.60) | 3879 (93.33) | 0.009 |
| Yes | | | | 110 (5.29) | 95 (4.57) | 231 (5.56) |  |
| Death | | | | 44 (2.12) | 38 (1.83) | 46 (1.11) |  |
| Congenital heart diseases | | | | | | | |
|  | |  | No | 1994 (95.96) | 2003 (96.39) | 4020 (96.73) | 0.027 |
|  | |  | Yes | 38 (1.83) | 33 (1.59) | 84 (2.02) |  |
|  | |  | Death | 46 (2.21) | 42 (2.02) | 52 (1.25) |  |
| Congenital anomalies- spinal cord and other nervous system defects | | | | | | | |
| No | | | | 2017 (97.06) | 2025 (97.45) | 4085 (98.29) | 0.027 |
| Yes | | | | 13 (0.63) | 12 (0.58) | 15 (0.36) |  |
| Death | | | | 48 (2.31) | 41 (1.97) | 56 (1.35) |  |
| Children, premature birth | | | |  |  |  |  |
| Pregnancy ≥ 37 week | | | | 1590 (76.52) | 1695 (81.57) | 3420 (82.29) | <0.001 |
| Pregnancy <37 week | | | | 488 (23.48) | 383 (18.43) | 736 (17.71) |  |
| Children, low birth weight | | | |  |  |  |  |
| ≥2500g | | | | 1584 (76.23) | 1676 (80.65) | 3529 (84.91) | <0.001 |
| <2500g | | | | 494 (23.77) | 402 (19.35) | 627 (15.09) |  |
| Mother, Heroin recidivating | | | |  |  |  |  |
| During pregnancy | | | | 1027 | 0 | 0 |  |
| Before pregnancy | | | | 902 | 991 | 0 |  |
| Mother, Amphetamine recidivating | | | | | | | |
| During pregnancy | | | | 1106 | 0 | 0 |  |
| Before pregnancy | | | | 895 | 1168 | 0 |  |
| Mother, Ketamine recidivating | | | |  |  |  |  |
| During pregnancy | | | | 153 | 0 | 0 |  |
| Before pregnancy | | | | 71 | 81 | 0 |  |
